# Brain Abscess Caused by Nocardia farcinica in a Young Immunocompetent Patient

**DOI:** 10.7759/cureus.40823

**Published:** 2023-06-22

**Authors:** Tetyana Okan, Saliman Esmati, Homayoon Lodeen, Michael Abawkaw, Jashandeep Kaur, Kaushik Doshi, Md Aticul Islam

**Affiliations:** 1 Department of Internal Medicine, Jamaica Hospital Medical Center, New York, USA; 2 Department of Internal Medicine, New York Institute of Technology College of Osteopathic Medicine, Old Westbury, USA; 3 Department of Infectious Diseases, Jamaica Hospital Medical Center, New York, USA

**Keywords:** ct and mri brain, brain abscess excision, intravenous drug use (ivdu), allergic reaction to bactrim, brain abscess mri, non-immunocompromised, immunocompetent patients, cerebral nocardiosis, brain abscess, nocardia farcinica

## Abstract

Cerebral nocardiosis is a rare opportunistic infectious disease that occurs mainly in immunocompromised hosts; however, immunocompetent patients may be affected too. It often results in the formation of intraparenchymal brain abscess, which represents only 2% of all cerebral abscesses. The overall mortality rate exceeds 20% in immunocompetent patients and 55% in immunocompromised patients. Bacteriological diagnosis is often confirmed only after the surgical excision of the abscess. Thus, the initiation of effective therapy is frequently delayed. Our goal is to highlight a diagnostic approach to cerebral nocardiosis in an immunocompetent patient with the purpose of accelerating the initiation of the appropriate therapy. We report a rare case of brain abscess caused by *Nocardia farcinica *in a 39-year-old male, a resident of New York City, USA, with a past medical history of intravenous (IV) drug use, who was admitted for altered mental status. The patient was cachectic and ill-appearing. Initial laboratory tests showed neutrophilic leukocytosis. Computed tomography (CT) of the head revealed a large ill-defined multilobulated mass of size 6 × 5 × 4.5 cm in the right cerebral hemisphere, which was confirmed with magnetic resonance imaging (MRI). The hospital course was complicated by the deterioration of mental status requiring endotracheal intubation. The patient underwent a right-sided hemicraniectomy; a wound culture identified *Nocardia farcinica*. The patient was started on intravenous (IV) Bactrim, which caused an allergic reaction. Thus, he was switched to IV imipenem-cilastatin. After E-test was performed, the patient was switched to oral linezolid. The initiation of targeted antibiotic therapy was crucial for the management of this patient and resulted in a good clinical outcome. In conclusion, cerebral nocardiosis, being an unusual and a potentially fatal infection, should be considered in the differential diagnosis of brain abscess even in immunocompetent hosts. Prompt bacteriological diagnosis helps to initiate a specific antimicrobial therapy. Long-term antimicrobial therapy and long-term follow-up are necessary to prevent relapse.

## Introduction

Cerebral nocardiosis is a rare opportunistic infectious disease that occurs mainly in immunocompromised hosts [1-3]. However, immunocompetent patients may be affected too. Less than 35% of patients are immunocompetent at the time of diagnosis [4]. Cerebral involvement often leads to the formation of intraparenchymal brain abscess, which represents only 2% of all cerebral abscesses [2]. The overall mortality rate is high, exceeding 20% in immunocompetent patients and 55% in immunocompromised patients [1,2]. The diagnosis of this disease is seldom based on imaging. Bacteriological diagnosis is challenging and is often confirmed only after the surgical excision of the abscess [2]. Thus, initiating effective therapy is frequently delayed. We report a rare case of a brain abscess caused by *Nocardia farcinica* in a non-immunocompromised host. Our goal is to increase awareness among practitioners about this challenging diagnosis and to highlight a diagnostic approach to cerebral nocardiosis in an immunocompetent patient with the purpose of accelerating the confirmation of the diagnosis and, therefore, initiating the appropriate therapy early.

## Case presentation

A 39-year-old male, a resident of New York City, USA, with a past medical history of intravenous (IV) drug use, methadone replacement therapy, and cervical spine surgery due to methicillin-sensitive *Staphylococcus aureus* vertebral osteomyelitis was brought in by emergency medical services after he was found on the tracks at a subway station and was admitted with altered mental status. He was alert but uncooperative. He denied loss of consciousness, dizziness, visual changes, shortness of breath, cough, fever, rash, abdominal pain, nausea, or vomiting. In the past medical history, the patient had two documented admissions: Four months ago, he was admitted for altered mental status requiring intubation for airway protection secondary to serotonin syndrome, and eight months ago, he presented with severe otitis media. No pathogens were identified during previous hospitalizations. The patient was a tobacco and marijuana smoker and alcohol and intravenous drug (cocaine) user. However, he denied recent drug use. Initial vital signs were as follows: temperature, 37.0°C; blood pressure, 135/95 mmHg; heart rate, 62 beats per minute; and respiratory rate, 18 breaths per minute. His weight was 40.8 kg, BMI 14.09 kg/m^2^, and oxygen saturation (SpO_2_) 99% on room air. On physical examination, the patient was cachectic and ill-appearing with poor dentition. The cardiovascular and pulmonary systems were without peculiarities. Mentally, he was alert and oriented only to person but not to place or time. His cranial nerve and sensory examination was grossly intact bilaterally. His motor examination was as follows: left upper and lower extremities, 4/5; right upper and lower extremities, 5/5. Initial laboratory results showed neutrophilic leukocytosis with leukocytes of 14.6 × 10^9^/L (normal range: 4.0-11.0 × 10^9^/L) and neutrophils of 80% (normal range: 47%-74%), hemoglobin of 14.3 g/dL (normal range: 14-18 g/dL), mean corpuscular volume (MCV) of 85.5 fL (normal range: 80-100 fL), hematocrit of 41.9% (normal range: 41%-50%), platelets of 407 × 10^3^/L (normal range: 150-450 × 10^3^/L), sodium of 138 mEq/L (normal range: 135-145 mEq/L), potassium of 3.8 mEq/L (normal range: 3.5-5.2 mEq/L), chloride of 95 mEq/L (normal range: 95-106 mEq/L), carbon dioxide (CO_2_) of 32 mEq/L (normal range: 23-29 mEq/L), blood urea nitrogen (BUN) of 23 mg/dL (normal range: 6-20 mg/dL), creatinine of 0.7 mg/dL (normal range: 0.7-1.3 mg/dL), and normal liver function test.

**Figure 1:**
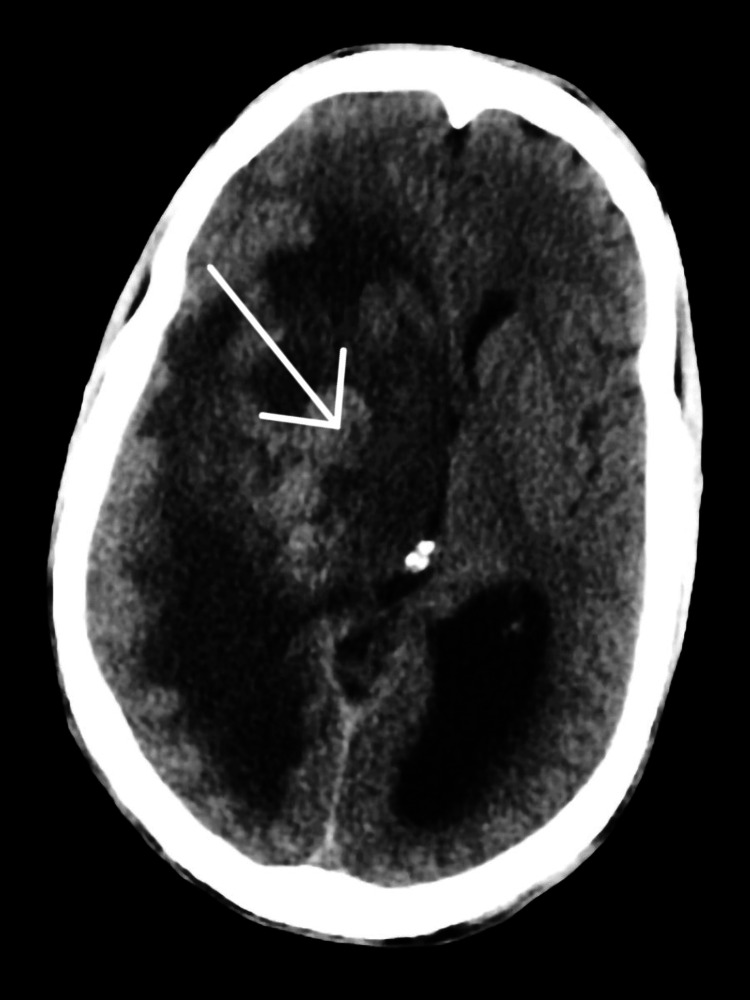
CT of the head. Large ill-defined multilobulated mass was revealed in the right cerebral hemisphere. CT: computed tomography

Computed tomography (CT) of the head was performed on the same day and revealed a large ill-defined multilobulated mass of size 60 × 50 × 45 mm in the right cerebral hemisphere with local extension from the temporal lobe to the right posterior gangliocapsular region and right thalamus (Figure 1), heterogeneous attenuation suggesting complex mixed cystic component of mass, secondary prominent vasogenic edema with compression of ventricles, and mass effect resulting in right to left subfalcine midline shift of 10 mm.

CT of the chest showed pulmonary emphysema with nonspecific bilateral lung scarring. CT of the cervical spine, abdomen, and pelvis did not reveal any evidence of metastatic disease.

The patient was admitted to a medical intensive care unit with large right cerebral mass, neutrophilic leukocytosis, and malnutrition. Differential diagnosis included primary central nervous system (CNS) malignancy, CNS lymphoma, metastatic disease, and brain abscess. Initial medical management included thiamine, folic acid, intravenous vancomycin and cefepime, metronidazole, intravenous dexamethasone, and Keppra. Methadone was resumed. Ondansetron, famotidine, and heparin were started. Additional laboratory tests were performed: Urine toxicology was positive for opiates; arterial blood gas showed respiratory alkalosis; comprehensive metabolic panel demonstrated hypokalemia, hypernatremia, mild hyperbilirubinemia, normal magnesium, and low phosphate (due to refeeding syndrome); and thyroid-stimulating hormone was normal. Hepatitis panel was positive for hepatitis C antibody; thus, additional tests for hepatitis C genotype and RNA were performed. Hepatitis B surface antigen, human immunodeficiency virus (HIV) antibody, and QuantiFERON tests were negative. No pathology was revealed on transthoracic echocardiography. Electrocardiogram demonstrated sinus bradycardia with occasional premature atrial contractions and prolonged QT interval of 470 ms, probably due to methadone replacement therapy.

**Figure 2:**
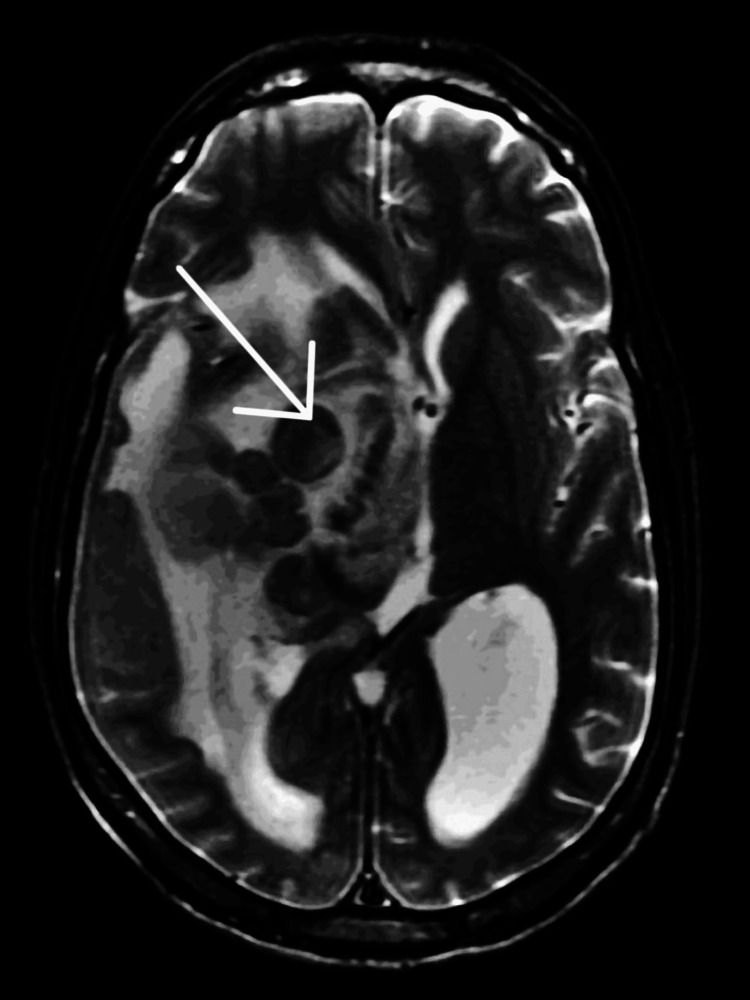
MRI of the brain. The multiloculated lesion in the right temporal lobe with extensive adjustment edema with mass effect and midline shift was confirmed. MRI: magnetic resonance imaging

Magnetic resonance imaging (MRI) of the brain with and without contrast was performed on the next day and confirmed a multiloculated lesion of size 47 × 47 × 52 mm in the right temporal lobe and right basal ganglia with intense peripheral enhancement and extensive adjustment edema with mass effect on the right midbrain, partial compression of the right lateral ventricle contributing to 12 mm midline shift, and right uncal herniation (Figure 2), concluding more likely an infectious process (abscess) than neoplasm.

**Figure 3:**
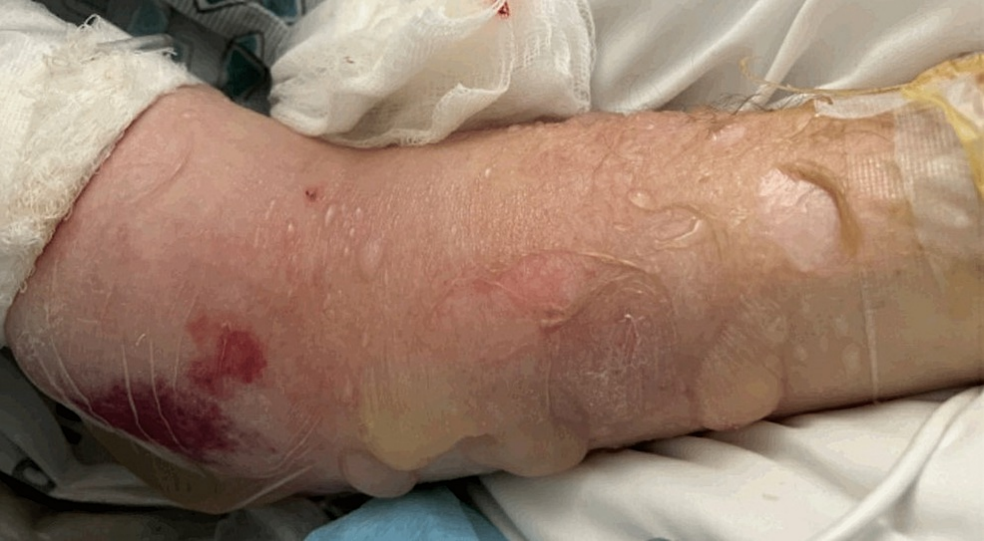
Allergic reaction to Bactrim. The figure shows multiple clear fluid-filled bullae on the left upper extremity, suggestive of Stevens-Johnson syndrome.

The hospital course was complicated by deteriorating mental status requiring endotracheal intubation for airway protection. The patient underwent a right-sided hemicraniectomy for decompression on day 4 of hospitalization. The brain abscess was resected, and cultures were taken. The patient was given broad-spectrum antibiotics. Mental status improved, and he was extubated after surgery. Repeated CT of the head on day 3 after surgery showed expected postsurgical changes, resided vasogenic edema, and mass effect, and midline shift improved to 6 mm. Wound culture identified *Nocardia farcinica*. Infectious disease specialist initially started Bactrim, while previous antibiotics were discontinued. However, Bactrim caused an allergic reaction in the form of multiple clear fluid-filled bullae in the left upper extremity, suggestive of Stevens-Johnson syndrome (Figure 3). Thus, he was switched to imipenem-cilastatin, and the dose was adjusted according to kidney function. The course of IV antibiotics lasted for six weeks.

In six weeks, leukocytosis resolved; the patient clinically improved and regained strength in the left upper and lower extremities. The decision was made to switch to oral antibiotics. As the duration of antibiotics was going to be prolonged (nine months to one year), an E-test was performed to detect sensitivity to oral antibiotics, which showed sensitivity to linezolid. Therefore, the patient's antibiotics were switched to oral linezolid.

The patient's condition was stable for discharge to shelter and follow-up with primary care practitioner, infection diseases, and neurology team outpatient. Additionally, the patient was followed up for hepatitis C treatment.

## Discussion

*Nocardia* species, filamentous aerobic gram-positive bacteria that belong to the order Actinomycetes, are found worldwide in soil and water. In addition, they are oral microflora found in healthy gingiva and periodontal pockets. They can cause localized or systemic disease in humans, who have preexisting immune system compromise. Patients with HIV, diabetes, cancer, lupus, and inflammatory bowel disease are at higher risk for infection [3,4]. In our case, the patient was a 39-year-old male with no immunodeficiency disease. However, taking into consideration his medical history of intravenous drug use, vertebral osteomyelitis, two recent hospitalizations, and antibiotic use together with poor dentition and cachexia, we may conclude that those factors were the key reasons for the patient's *Nocardia* infection.

A review published in 2020 found that the USA, India, and Mexico have the highest number of reported cases of nocardiosis in the literature [1]. Among those reported cases, 13 medically important species were identified [5]. *Nocardia asteroides*, *Nocardia farcinica*, *Nocardia nova*, and *Nocardia abscessus* cause the majority of invasive infections [5]. They commonly affect the lung, brain, and skin and less frequently the kidneys, spleen, liver, bone, and joints. *Nocardia farcinica* is more frequently associated with central nervous system (CNS) involvement than other species [2].

The clinical presentation of CNS nocardiosis is variable, and there are no specific symptoms to make the diagnosis. The patients may present with headaches, seizures, focal neurological abnormalities, and altered mental status. Other possible symptoms include fever, weight loss, and night sweats [4]. The clinical manifestations of CNS nocardiosis usually result from local effects of granulomas or abscesses in the brain; less commonly, the lesions are found in the spinal cord or meninges [2]. The average age of infection onset is 40, and infections typically occur more often in males than in females [5]. 

To make a diagnosis of CNS nocardiosis, a full patient history should be obtained along with proper neurological imaging. The imaging should include CT of the head and MRI of the brain. Abscesses that are larger than 25 mm in diameter and that fail to shrink after four weeks of antibiotic therapy should be aspirated to confirm the diagnosis regardless of the immune status of the patient [2]. However, aggressive craniotomy must be considered for the patients with systemic infection and multiple brain lesions. The bacteriological confirmation of the diagnosis is often needed. Since the clinical and radiologic manifestations are nonspecific, the microbiological diagnosis is often challenging due to the difficulties and slowness of culture growth, as well as the lack of a serologic test for nocardiosis; thus, CNS nocardiosis may be mistaken for other diseases (CNS malignancy, metastatic disease, or CNS lymphoma) [3]. CNS nocardiosis should be included in the differential diagnosis of brain abscess for both immunocompromised and immunocompetent patients [3].

Trimethoprim/sulfamethoxazole (TMP/SMX) is the treatment of choice, because of its ability to cross the hematoencephalic barrier. Other treatment options include amikacin, moxifloxacin, cephalosporins, linezolid, and imipenem-cilastatin [3,4]. Empiric therapy is usually initiated before antibiotic susceptibility tests return. *Nocardia farcinica* has increased antibiotic resistance to beta-lactams and most of aminoglycosides compared to other strains [4].

Therapy must be prolonged to prevent relapses. For patients with CNS involvement, therapy is usually continued for 6-12 months, and the patients are monitored thereafter for infection recurrence [5]. In addition, surgical management may be required, especially in the patients with severe disease and failure of antimicrobial therapy [4]. In our case, the patient required both antimicrobial therapy and surgical approach. The patient developed an allergic reaction to TMP/SMX and required another antibiotic.

High mortality rate is attributed to the severity of underlying disease, difficulties in identifying the pathogen, and its resistance to antibiotics, leading to the inappropriate or late initiation of therapy. *Nocardia farcinica* is usually difficult to treat and often requires a multidisciplinary approach.

## Conclusions

Cerebral nocardiosis, being an unusual and potentially fatal infection of the central nervous system, should be considered in the differential diagnosis of brain abscess, not only in immunocompromised patients but also in immunocompetent hosts. Early diagnosis is imperative to reduce the mortality rate and improve the quality of life of such patients. Thus, reasonable surgical intervention with a biopsy allows not only to reduce the mass effect symptoms but also to confirm the diagnosis. Prompt bacteriological diagnosis based on the culture of the excised abscess helps to initiate a specific antimicrobial therapy, which often leads to a good clinical outcome. Long-term antimicrobial therapy and long-term follow-up are necessary to prevent relapse. Additional studies are needed to improve the diagnostic approach and outcomes of this rare infectious disease.
